# Comparative Analysis of Structural Variations Due to Genome Shuffling of *Bacillus Subtilis* VS15 for Improved Cellulase Production

**DOI:** 10.3390/ijms21041299

**Published:** 2020-02-14

**Authors:** Soujanya Lakshmi Ega, Gene Drendel, Steve Petrovski, Eleonora Egidi, Ashley E. Franks, Sudhamani Muddada

**Affiliations:** 1 Department of Biotechnology, K L E F University, Guntur 522 502, India; soujanyabiochem@gmail.com; 2 Department of Physiology, Anatomy and Microbiology, College of Science, Health and Engineering, La Trobe University, Melbourne, Victoria 3086, Australia; 16118761@students.latrobe.edu.au (G.D.); steve.petrovski@latrobe.edu.au (S.P.); E.Egidi@westernsydney.edu.au (E.E.); A.Franks@latrobe.edu.au (A.E.F.); 3 Hawkesbury Institute for the Environment, Western Sydney University, Sydney, NSW 2750, Australia; 4 Centre for Future Landscapes, College of Science, Health and Engineering, La Trobe University, Melbourne, VI 3086, Australia

**Keywords:** *Bacillus subtilis* VS15, genome shuffling, cellulase, next generation sequencing (NGS), single nucleotide polymorphism (SNP)

## Abstract

Cellulose is one of the most abundant and renewable biomass products used for the production of bioethanol. Cellulose can be efficiently hydrolyzed by *Bacillus subtilis* VS15, a strain isolate obtained from decomposing logs. A genome shuffling approach was implemented to improve the cellulase activity of *Bacillus subtilis* VS15. Mutant strains were created using ethyl methyl sulfonate (EMS), *N*-Methyl-*N*′ nitro-*N*-nitrosoguanidine (NTG), and ultraviolet light (UV) followed by recursive protoplast fusion. After two rounds of shuffling, the mutants Gb2, Gc8, and Gd7 were produced that had an increase in cellulase activity of 128%, 148%, and 167%, respectively, in comparison to the wild type VS15. The genetic diversity of the shuffled strain Gd7 and wild type VS15 was compared at whole genome level. Genomic-level comparisons identified a set of eight genes, consisting of cellulase and regulatory genes, of interest for further analyses. Various genes were identified with insertions and deletions that may be involved in improved celluase production in Gd7. Strain Gd7 maintained the capability of hydrolyzing wheatbran to glucose and converting glucose to ethanol by fermentation with *Saccharomyces cerevisiae* of the wild type VS17. This ability was further confirmed by the acidified potassium dichromate (K_2_Cr_2_O_7_) method.

## 1. Introduction

Lignocellulosic biomass is an abundant bio-renewable, carbon-neutral resource. It is the basis for alternative energy platforms with the potential to decrease CO_2_ emissions and atmospheric pollution by reducing the usage of fossil fuels [1]. Due to the abundance and availability of lignocellulosic biomass, lignocellulosic-derived cellulosic ethanol represents a significant and inexpensive petroleum fuel alternative [2]. Lignocellulosic derived glucose is a potential feed stock for a wide variety of biologically produced chemicals and bio fuels [3]. Cellulase has been widely used in various industrial applications such as the food, animal feed, beer and wine, textile, laundry industry pulp, paper, agriculture, bio-fuel, and pharmaceutical industries amongst others [4].

Lignocellulosic biomass comprises of three major components: cellulose, hemicellulose, and lignin. Cellulose can be hydrolyzed by a group of enzymes referred to as cellulases, which themselves are classified within carbohydrate-active enzymes (CAZymes), including exoglucanase, endoglucanase, and β glucosidase [5]. Endoglucanases, or Endo-1, 4-β-d-glucanase (EC 3.2.1.4), act on amorphous sites. cleave randomly at internal glycoside bonds to generate both reducing and non-reducing ends of cellulose. Exoglucanase, or 1,4-β-d-cellobiohydrolase (EC 3.2.1.91), hydrolyzes the reducing or non-reducing ends of cellulose, liberating either cellobiose or glucose as the major products. β-glucosidase (EC 3.2.1.21) is a key enzyme for complete hydrolyzing of cellobiose to liberate free glucose molecules [5].

Increases in cellulase production have been achieved through a number of different approaches such as classical strain improvement (CSI), metabolic engineering, and synthetic biology [6]. While CSI is robust, it is also time consuming, laborious, and depends on either mutagenesis followed by phenotypic screening for improved characteristics, or manipulation of desired genes known to play a significant role in the desired phenotype [7].

Genome shuffling is an efficient tool to generate improved strains, whereby the genome of multiple parent strains is shuffled through recursive recombination. This can be achieved by multi-parental crossing though DNA shuffling with a homologous recombination of entire genomes, analogous to conventional breeding [8]. Through this process, microbial genome shuffling efficiently generates combinatorial libraries of new strains with diverse genetic potential. Hence, this approach is able to produce strains with improved characteristics and efficiencies in respect to desirable phenotypes. For example, genome shuffling improved the production of Tylosin by *Streptomycin fradie,* which has been estimated to reduce an approximately twenty year and a one million strain screen classical approach to just a year and 24,000 screens [9].

Over the past decade, applications of genome shuffling have extended to various sectors, such as the health care, pharmaceutical, food, agriculture, textile and chemical industries. It has been used to enhance microbial stress and acid tolerance, as well as antibiotic production. The greatest advantage of genome shuffling is that many genes and their regulatory units can be randomly changed throughout the entire genome without prior knowledge of genome sequence information [10]. Initially this method was developed for bacteria and was later extended to yeast and fungi. For example, reports using genome shuffling include increased antifungal activity in *Lactobacillus plantarum* [11], increased acetic acid tolerance of ethanologenic yeast *Candida krusei* GL560 [12], and increased production of cellulase production in *Trichoderma viride* [13].

Although fungi are the common sources of cellulases, cellulases from other microorganisms are being explored [14,15]. This is important because of the varied applications of cellulases in diverse backgrounds (paper, textiles, food, etc.). The diversity of microorganisms can cater to the varied conditions prevailing in different industrial applications. Further, the efforts to reduce cost and improve efficiency require better enzymes. Members of *Bacillus sp.* are known to produce cellulases [14,16,17]. They may be advantageous because of their fast growth. Further, the genomes are simple and convenient to manipulate.

*B. subtilis* is known to possess numerous CAZymes (www.cazy.org; File S3), with the *B. subtilis* subspecies spizizenii str.W23, *B. subtilis B. subsp.* natto BEST195, and *B. subtilis subsp* subtilis str.168 having between 125 and 145 [18,19]. *B. subtilis* has also been utilized as a source for endogluconases, and thermostable β-glucosidases, with potential for improving bioethanol production [20,21]. Any mutations in the structural genes for cellulase activity or other CAZymes may influence the cellulase activity.

In this study, .genome shuffling was chosen to enhance the cellulase production in *Bacillus subtilis* VS15. Subsequently, the genetic variation between the wild VS15 and the mutant Gd7 strains was identified by using whole genome sequencing. We have sequenced assembly and annotated the whole genomes of both wild and mutant strains (VS15 and Gd7), and performed the comparative genome analysis with reference strain *Bacillus subtilis subsp. subtilis* str. 168 as a model cellulase producer.

The present study is a first attempt to improve the cellulase production in *Bacillus subtilis* strains through genome shuffling and comparative analysis between improved and wild strain by using whole genome resequencing.

## 2. Results 

### 2.1. Screening for Cellulase Overproducing Mutant Strains

In order to produce mutants, mutagens EMS and NTG with UV were utilized. Various doses of the mutagens EMS (0.620–1.48 mM), NTG (2–5 µg) grains and UV (1–10 min) were tested to find out the optimum dose. Optimal rates were observed at 0.931 mM of EMS, 2 µg grains of NTG, and exposure to UV for 3 min, respectively.

After mutagenesis by the combination of EMS and UV, a total of 400 mutants were evaluated for increased cellulase production. Three mutants with increased cellulase production: EUA9, EUB8, and EUD6, were selected for further study. Similarly, after mutagenesis by NTG and UV mutagens, 298 colonies were evaluated, and NUA7, NUB2, and NUD8 were selected for recursive protoplast fusion. All six mutants had a slight increase in cellulase production in the range of 3.77–5.9 IU/mL compared to the wild type (3.77 IU/mL) referred to in Table 1.

### 2.2. Genome Shuffling by Recursive Protoplast Fusion

Protoplast preparation efficiency was enhanced to 70% by the addition of lysozyme in the protoplast buffer. Inclusions of Mutanolysin in the protoplast buffer further increased the frequency of protoplast formation to 92%.

**Figure 1:**
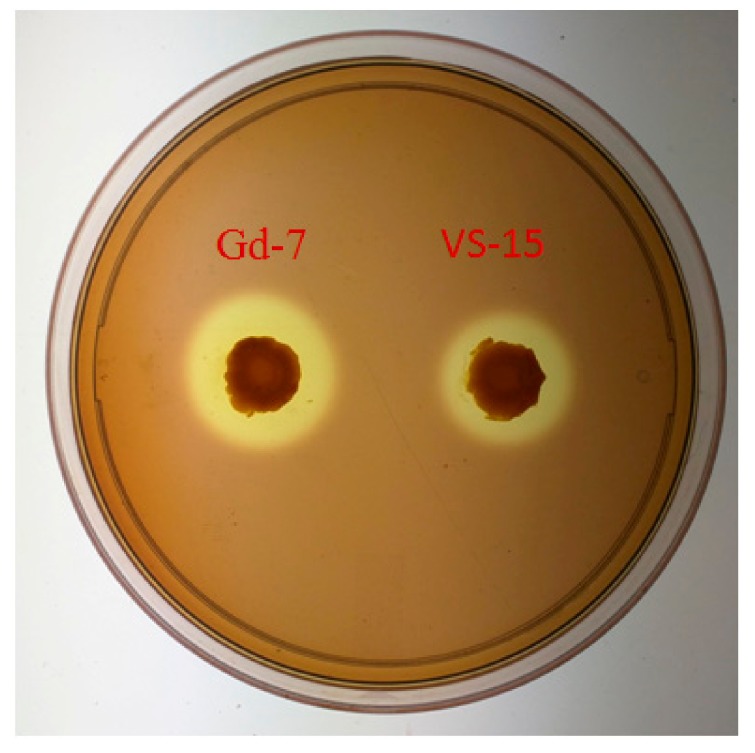
Comparison of wild strain VS15 and shuffled strain Gd7 on an agar plate containing 2.5% caroxymethylcellulose (CMC, incubated for 16 h).

The six mutants with increased cellulase production (EUA9, EUB8, EUD6, NUA7, NUB2, and NUD8) were employed for the first round of fusion. After the first round of shuffling, ninety colonies were screened for faster growth and bigger zone of hydrolysis. The shuffled strains G1, G21, G24, and G56 had CMCase activity of 7.27 IU/mL, 7.69 IU/mL, 7.91 IU/mL, and 8.06 IU/mL with an enhancement of 93%, 104%, 110%, and 114%, respectively, over the wild type (3.77 IU/mL). The increase in the zone of hydrolysis ranged from 2.2–2.5cm. Four isolates were selected for a second round of genome shuffling. After the second round of genome shuffling, 50 isolates were screened. The colonies Gb2, Gc8, and Gd7 showed CMCase activity of 8.9 IU/mL, 9.5 IU/mL, and 10.08 IU/mL corresponding to a 136%, 151%, and 167% enhancement over the wild type, respectively, with a zone of hydrolysis ranging from 3.15 cm to 3.5 cm (Figure 1). 

**Figure 2:**
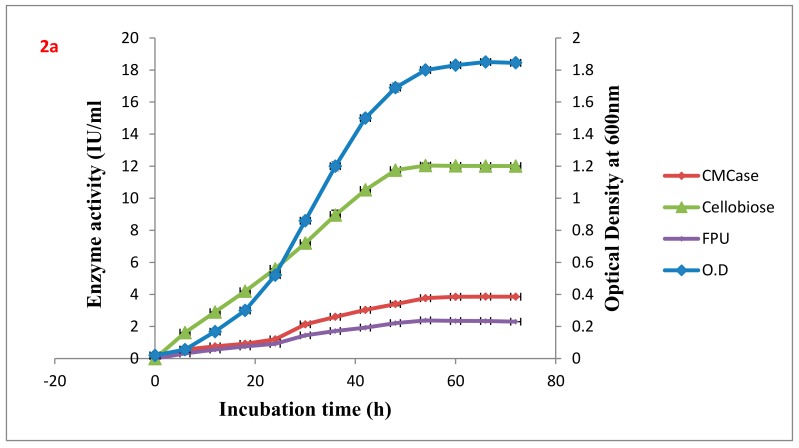

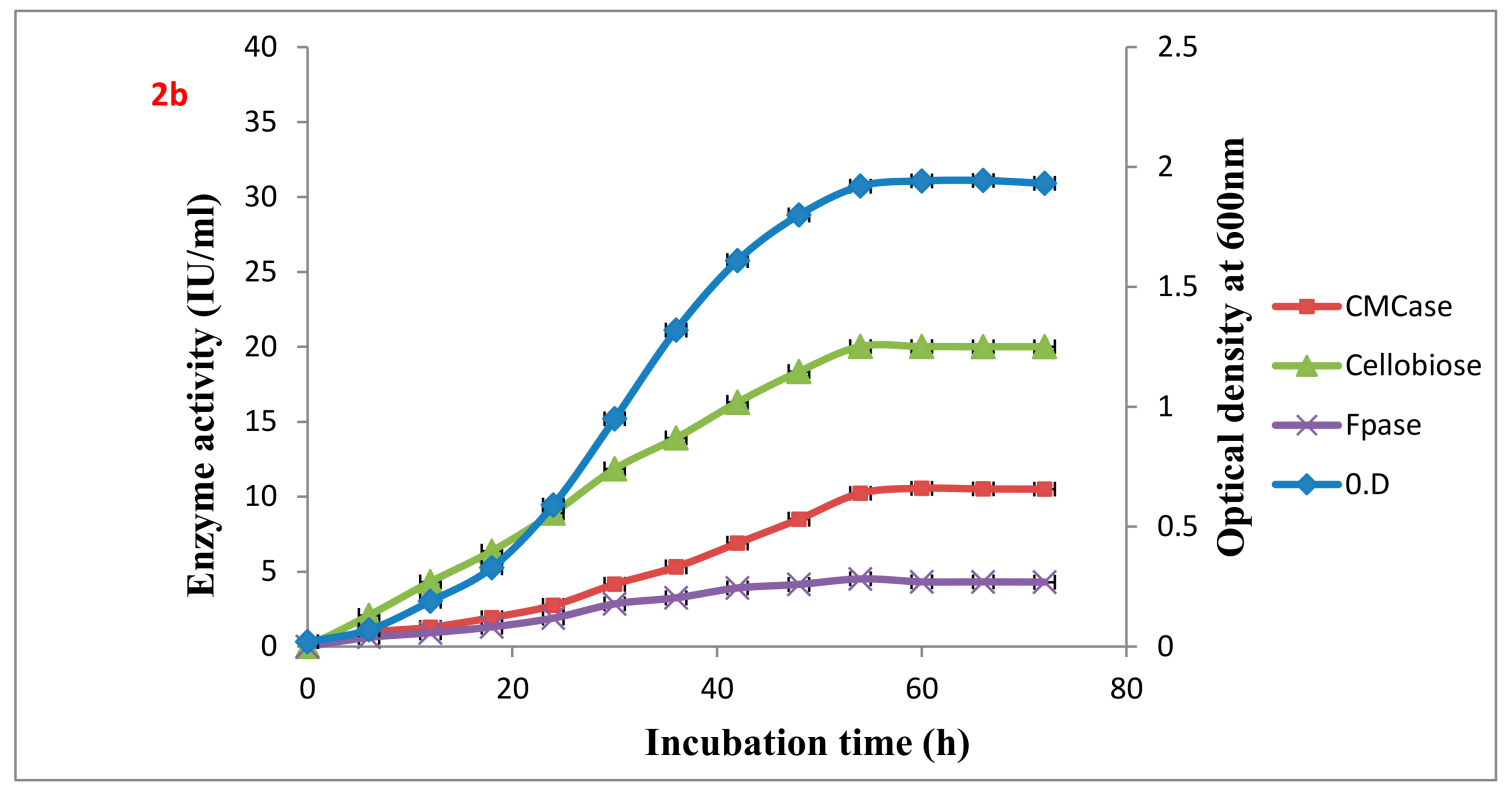
Growth curve and production of CMCase, cellobiose, and FPase (**a**) VS15 (**b**) Gd7.

The efficiency of improved strains utilization of other carbon sources was analyzed using filter paper, carboxy methyl cellulose, cellobiose, and cotton gin waste. Supernatants from cultures grown for 54 h on an 8% wheat bran medium in a shake flask had 53–75%, 136–167%, 60–96% and 20–40% enhancement in FPase, CMCase, cotton gin waste, and Cellobiose assays compared to the wild type strain. Activities of FPase, CMCase, cotton gin waste, Cellobiose, and extracellular protein concentrations of the shuffled strains were higher than that of the wild types, as shown in Table 2. The wild type VS15 and the highest producer of all the fusants Gd7 were analyzed for CMCase, FPase, and cellobiase production with respect to the growth curve in an 8% Wheat bran medium at 37 °C (Figure 2a,b).

### 2.3. Utilization of Cotton Gin Waste as a Substrate

Acid pre-treatment increased enzymatic hydrolysis and sugar release of the cotton ginning waste. Initially, an increase in acidic concentration resulted in higher sugar releases from 8.93 IU/mL at 1% to 11.08 IU/mL at 3%. A further increase in the acid concentration past 3% caused decreases in the release of sugars to 7.56 IU/mL at 5% and 7.43 IU/mL at 7%. The optimum acid concentration for the pre-treatment was set at 3%. Gb-2, Gc-8 and Gd-7 isolates were used. At 3% concentration, acid showed 9.05 IU/mL, 9.78 IU/mL, and 11.08 IU/mL activity with an enhancement of 60%, 73%, and 96%, respectively, when compared to that of wild types (5.654 IU/mL) on cotton gin waste (Table 2).

### 2.4. Hydrolysis Activity of VS 15 and the Shuffled Strains on the CMC Agar Plates 

Zone of clearance and colony diameter was measured by using Image J software after 16 h of incubation. Shuffled strains Gb2, Gc8, and Gd7 showed a maximum zone of hydrolysis in the range of 232–244 cm^2^ around the colony, with a diameter in the range 79–89 cm^2^, an approximately 1.5 fold increase over the wild type zone of hydrolysis of 121 cm^2^ and colony diameter of 52 cm^2^.

### 2.5. Bioethanol Production

Previous studies reported that through the action of cellulase enzyme, bioethanol production was achieved involving simultaneous mechanism of fermentation and saccharification with saccharomyces cerevisiae [22]. In this study, the fermentation of VS15 and Gd7 in 8% wheat bran medium and saccharification with 1% *saccharomyces cerevisae,* converts the cellulose into ethanol. Further, the ethanol production was confirmed by the K_2_Cr_2_O_7_ method. Through the reaction of K_2_Cr_2_O_7_ with these samples, it was observed that a color change occurred from yellow-orange to green blue. These significant results showed that *saccharomyces cerevisae* utilized the liberated sugars from cellulose degradation and show that *Bacillus subtilis* VS15 and Gd7 strains are capable of fermenting wheat bran to facilitate the production of biofuel. 

### 2.6. Whole Genome Sequence

Whole Genome sequence data is deposited at DDBJ/ENA/GenBank for the strains VS15 and Gd7 under the accession numbers QFZO00000000 and MSEJ00000000, respectively. The versions described in this paper are QFZO01000000 and MSEJ01000000. 

### 2.7. Assembly and Annotation

No putative misassembles were detected with this genome assembly. The genome size of *Bacillus subtilis* VS15 strain is 4,163,202 bp, and the mean GC content is 43.71%, with a final number of contigs of 67 and an N50 value of 293,644 bp (Table 3). The genome assembly size of Gd7 is 4,146,024 bp, and the mean GC content is 43.65%, with a final number of contigs of 63 and an N50 value of 1,023,553 bp.

All assemblies were annotated using online RAST (Rapid Annotations using Subsystems Technology) Server to evaluate gene set completeness [23]. A total of 4426 and 4375 genes were predicted for VS15 and Gd7, respectively (Table 4). Out of 4426 genes, 4316 protein coding gene was reported for VS15, which includes 3317 genes with characterized protein annotation and 1038 hypothetical/putative proteins. Out of 110 predicted non-protein coding genes, 84 are rRNA encoding genes and the remaining 26 codes are for tRNA.

Whereas for Gd7 assembly, out of 4375 predicted genes, 4278 were annotated as protein coding genes and 97 are non-protein coding genes. Altogether, 3287 genes have a characterized protein and the remaining 1017 genes are hypothetical/putative proteins. Out of a total of 97 non-coding genes, 79 genes code for rRNA and 18 code for tRNA.

RAST identified 397 carbohydrate metabolism encoding enzymes. Among these enzymes, 25GHs, 4CEs, 5GTs, 2PLs, and 0AAs were identified as CAZymes using the CAZy database (File S2).

#### 2.7.1. SNP Calling

Using *Bacillus subtilis subsp. subtilis* str. 168 as reference strains, a total of 40166 SNPs and 758 indels were reported by VarScan for VS15, and 45797 SNPs and 1104 indels were reported for a Gd7 sample with a minimum coverage of 8, minimum variant frequency of 0.2, minimum average quality of 15, and a *P*-value threshold ≤ 0.01.

**Figure 3:**
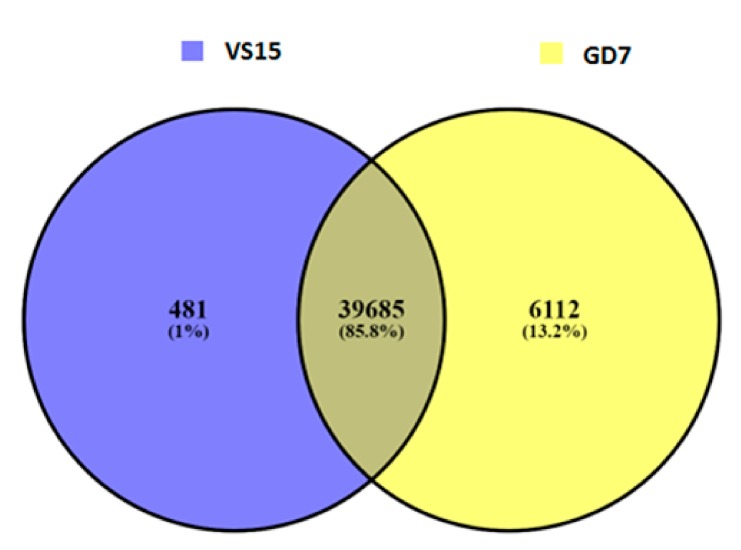
Venn diagram showing position-wise common and unique SNPs of VS15 and Gd7.

**Figure 4:**
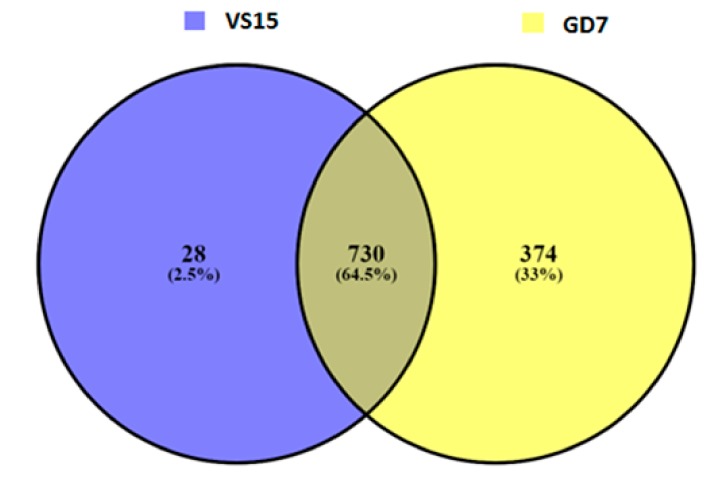
Venn diagram showing position-wise common and unique Indels of VS15 and Gd7.

**Figure 5:**
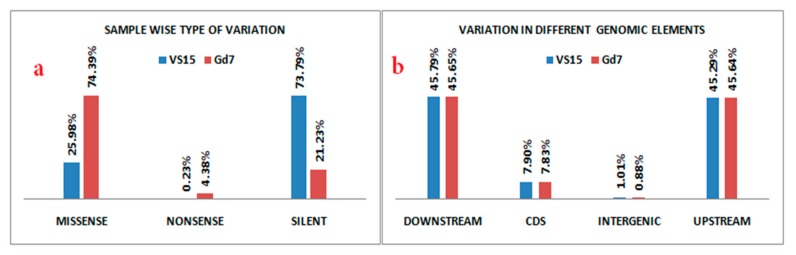
(**a**) Type of variation of the basis of effect; (**b**) Distribution of effect of variation in different genomic regions.

A total of 4375 and 4358 genes were affected by variation i.e., both SNPs and indels in VS15 and Gd7, respectively. Out of total 4375 affected genes, 4194 are protein coding genes, 80 rRNA, 66 pseudogenes, 27 sRNA, 6 lincRNA, 1 antisense, and 1 ribozyme genes. Out of total 4358 affected genes in Gd7, 4176 genes are protein coding, 80 rRNA, 67 pseudogenes, 27 sRNA, 6 lincRNA, 1 antisense, and 1 ribozyme gene. The effect of variation in both the samples is more abundant in RNA encoding genes. 481 unique SNPs were found in VS15, whereas in Gd7, 6112 unique SNPs were found, which were represented in a Venn diagram (Figure 3). A total of 28 unique indels were predicted for sample VS15 and 374 unique indels were predicted by DELLY in Gd7 (Figure 4). SNP variation in VS15 and Gd7 were performed on the basis of effect and its distribution in different genomic regions (Figure 5a,b).

#### 2.7.2. Repeat Analysis

A total of 235 and 133 SSRs were predicted by FullSSR for VS15 and Gd7, respectively. VS15 was predicated to have 85 inverted repeats by using the inverted utility of the EMBOSS package. Out of the 85 inverted repeats, 26 showed inversion with 100% identity, 13 with 94%, and 9 with 95% identity. Gd7 had 25 inverted repeats showing 100% inversion identity, 12 with 94%, and 10 with 95% inversion identity out of a total of 81 inverted repeats. There were 32 and 35 tandem repeats predicted for the VS15 and Gd7 samples, respectively, as well as 1492 and 1483 palindromic sequences with zero mismatches for sample VS15 and sample Gd7. The average size of palindromic sequence for both samples is 11 bp. A total of 9066 total direct repeats were predicted by Red in sample VS15 with a 263 bp average repeat length. In sample Gd7, 8548 direct repeats have been predicted with the average length of 286 bp.

### 2.8. Specific Gene Set Analysis for Single Nucleotide Polymorphisms (SNPs)

A total of eight genes playing a key role in cellulase production were selected. Four genes (*bglA, bglC* and *bglH* and *EglS*) cause hydrolysis of lignocellulosic biomass. BGL family genes are β-glucosidases, which hydrolyze the non-reducing end β-D-glucosyl residues to release glucose. Among the three BGL family genes (*bglA, bglC,* and *bglH*), one unique mutation was observed in the bglH gene region at position 4033186 (A substituted with G) compared to its wild type. However, no change in the translated message could be found (Figure S3). EglS is a hydrolytic enzyme which hydrolyzes the β (1–4) d-glucosidic linkage. A unique mutation in Gd7 was identified at position 1941335 (changing base A to G). This mutation does not affect the translated messenger RNA or repress the gene function.

Three genes are involved in the regulation of cellulase production. Regulation is mainly caused by carbon catabolite repression (CCR) and LicT anti-termination. The antiterminator LicT specifically binds to the genes involved in cellulase production. LicT positively regulates *bglC* gene [24]. The absolute binding position of *LicT* on *bglC* genes is located in the region starting from 4035760 to 4035792 with +25 bp upstream and +57 bp downstream, and the binding sequence/cis-element is GGATTGTTACTGCGAAAGCAGGCAAAACCTAAA. Four SNPs are associated with the binding range of LicT on bglC. Out of these four SNPs, three were located within additional +54bp downstream to the absolute binding position, whereas one SNP occurred at 4,035,780 bp, within the actual LicT-bglC binding site (changing base G to A). Although we did not find any variation in the gene expression of the active gene, these mutations may indirectly influence the enhanced cellulase production.

The CCR mechanism in *Bacillus subtilis* is controlled by CcpA and HprK phosphorylation. Sugar transportation is initiated by entry of glucose via the cytoplasmic membrane (CM) along with the EIICBA^Glc^ component, which provides phosphorylation to the received glucose. The resultant glucose phosphate (Glc-P) activates Hpr that phosphorylates at two sites, serine and histidine. At the serine regulatory site, Hpr is phosphorylated as Hpr-S_46_-P, with the consumption of ATP. Later, Hpr-S_46_-P couples with regulatory protein CcpA. The Hpr-S_46_-P-CcpA complex binds to DNA at the cre (catabolite responsive element) site in the promoter region of the target gene that represses the transcription of the gene encoding hydrolytic enzymes (cellulase, xylanase etc.). In our study, within the CcpA region, nine SNPs were identified in Gd7, while six SNPs were identified in VS15, when the genome of those strains was compared to the reference strain *Bacillus subtilis subsp. subtilis* str. 168. Three unique mutations were identified in the mutant Gd7 at positions 3044276 (G to A), 3044348 (A to G), and 3044930 (C to T) compared to its wild type.

In *Bacillus subtilis,* Hpr is a signaling intermediate which is phosphorylated by HprK. Eight SNPs were found in VS15, whereas eleven SNPs were found in Gd7 compared to the reference strain *Bacillus subtilis subsp. subtilis* str. 168. Among these eleven SNPs, three unique mutations were observed in the mutant (Gd7) at positions 3594559 (C to T), 3595004 (T to C), and 3595028 (C to T) compared to its wild type (VS15). However, no change in the amino acid sequence was observed.

YxaL is a protein kinase enzyme, and repression or deletion of this enzyme results in various defects in glucose metabolism, which tends to force the cell under starvation condition and looking for the alternative source. This condition causes starved cells to switch to the production of hydrolytic enzymes and uptake of cellular storage . YxaL is important for the production of hydrolytic enzymes. Here we found three unique mutations at positions 4102465 (A to T), 4102474 (T to C) and 4102591 (A to C), although these mutations do not alter the translated message. 

The detailed SNP distribution among the specific gene set is shown in Table 5 and File S1a. 

### 2.9. Specific Gene Set Analysis for InDels

For the selected gene set, InDels for both VS15 and Gd7 samples were analyzed and insertions were found in all of the samples. Three InDels in sample VS15 (Table 6 and Table 7) and seven InDels in sample Gd7 (Table 8 and Table 9) were found compared to the reference strain *Bacillus subtilis subsp. subtilis* str. 168. These InDels have been found upstream within 5 kb of the gene. Among these seven InDels in Gd7, *BglC,* and *LicT* genes were affected three times, whereas *EglS*, gene were affected two times. In wild type (VS15) *BglC*, *EglS*, and *LicT* genes had 1 InDel each. In Gd7, seven InDels were observed, with four being unique InDels when compared to the wild type (VS15). A nucleotide insertion was observed in BglC at a position 369225 (G is inserted at reference base T and the altered base composition was TG) −1.034 kb upstream to the gene.

The *licT* gene had two insertions, one at a position 4011724 (GAG is inserted in the position T and the altered bases are TGAG) −1.142 kb upstream to the gene, and the other at the position 4011712 with three nucleotide insertions (ACA is inserted in the reference position T and the altered bases are TACA) −1.154 kb upstream to the gene.

A single nucleotide insertion in EglS was observed at position 1938628 (T is inserted in the reference position G and the altered base pair is GT) −1.997 kb upstream to the gene region. All these InDels did not alter the translated messenger RNA.

Besides Indels away from the orfs, Indels could also be located within genes also (Table 7 and Table 9). These deletions/insertions lead to frame shift in the reading frame of the ribosome leading resulting in altered translated sequence(as in fatR and GpsA; File S4) and can lead to altered expression of respective genes or loss of function. These altered expression of these genes possibly influence the cellulase expression.

## 3. Discussion

Genome shuffling enhanced cellulase production in *Bacillus subtilis* VS15 through the production of improved mutant strains. Mutagenesis using EMS with UV and NTG with UV produced starting strains for genome shuffling. Shuffled strains were then developed by pooling of desired traits through homologous recombination by protoplast fusion. In order to identify the mutations responsible for this enhanced production of cellulase, the whole genome of two strains (*Bacillus subtilis* wild type VS15 and mutant type Gd7) were sequenced by NGS and compared to *Bacillus subtilis subsp. subtilis* str. 168 as a reference genome.

After two rounds of genome shuffling, the CMCase, FPase and Cellobiose activity of Gd7 increased to 167%, 75%, and 40%, respectively, over that of wild type VS15 (Table 2). Previous studies [25,26,27] reported that the increased growth rate in mutants allows the cells to utilize nutrients more effectively than their respective wild type strains, suggesting that increased extracellular production of enzyme proteins may contribute to enhanced production of cellulase. Earlier genome shuffling studies reported improved FPase activity in *Penicillium decumbens* JU-A10 by 117%, 142%, and 118% by UV, EMS, and N+ ion implantation mutagenesis, respectively, after two rounds of shuffling and screening over 2000 shuffled strains [28]. Cellulase activity in *Trichoderma viride* F161 was enhanced by 1.97 fold using UV irradiation, low-energy ion beam implantation, and Atmospheric Pressure Non-equilibrium Discharge plasma (APNEDP) [13]. Improved FPase and CMCase activity was observed in *Aspergillus glaucus* HGZ-2 by 1.95 fold and 1.72 fold, respectively, through UV irradiation after two rounds of shuffling [29].

Cellulolytic enzymes were induced and repressed depending on the availability of easily utilizable sugars [14]. Cellulase inducers included sepharose, cellulose, cellobiose, δcellobiose-1-5, lactone, lactose, and other oxidized products of cellulose [30]. In *Trichoderma Reesei*, two CBH genes (*cbh1, cbh2*), five endoglucanases genes (*Eg1, Eg2*, *Eg3*, *Eg4*, and *Eg5*) [31,32] and two b-glucosidase (*bgl1/cel3a, bgl2/cel1a*) are reported as cellulase producer genes, and five positive transcriptional activators (XYR1, ACE II, HAP 2/3/5, PacC, and AreA) have been identified to play a major role in gene regulation and expression of cellulase. In addition, three repressors (ACE I, Rce1, and CRE I [33,34] have been identified. In *Penicillium oxalicum*, a novel transcription factor, ClrC, has been identified, which regulates the multiple stress responses and cellulase expression. The absence of ClrC in a mutant strain reduces the induction of cellulase expression [35].

Transcriptional activators play an important role in the regulation of the production of cellulase in fungal species. Two transcription factors (clr-1 and clr-2) from *Neurospora crassa* which were required for growth have been identified [36]. Phylogenetic analyses also confirmed that clr-1 and clr-2 are conserved in the genomes of filamentous ascomycete fungi capable of degrading cellulose, but is not required for either growth or hemicellulase activity on xylan. A deletion of the clr-2 homolog (clrB) transcription factor in *Aspergillus nidulans* failed to induce cellulase gene expression, and reduced cellulolytic activity on Avicel was observed [36]. 

As DBTBS [24] reported, the LicT will regulate the bgIP gene with positive regulation. In the Gd7, one SNP occurred at the actual binding site of bgl gene and three SNPs were observed between 4035735 bp and 4035849 bp compared to the reference strain. These mutations may influence Bgl expression.

The mutant GD7 was obtained by genome shuffling. In this regard, a number of mutations were observed (Table 5, Table 7 and Table 9, Figure 3, Figure 4 and Figure 5). The phenotype of enhanced cellulase production could be the result of these mutations. It may not be possible to attribute the activity to a single mutation and its effect. In this regard, the possible role of multiple mutations in a group of genes is proposed.

A mutant β-glucosidase (bgl) of *Spodoptera frugiperda,* obtained by random mutagenesis, increased the rate of the cellulase production [37]. In our study, a SNP was observed in bglH and eglS. However, these mutations may not lead to an affective expression of the cellulase gene since the translated messenger RNA was unchanged. Similarly, mutations in the yxaL gene do not seem to influence the cellulase production.

In bacteria, cellulase gene regulation is finely controlled by a regulatory mechanism through two key regulons: CcpA and LicT. Carbon Catabolite Repression (CCR) is referred as a glucose effect. The keys factors involved in the CCR are CcpA, Hpr protein, Hpr kinase/phosphorylase and glycolytic intermediates (G6p and FDP) [38]. In *Bacillus subtilis,* CCR is mediated by catabolite control protein A (CcpA). Bacteria can prefer the glucose as a primary carbon source, and it cannot utilize any secondary substrate by allowing the CcpA to block several catabolite genes and often also to repress the transcription of alternative sugar pathway operons. CcpA is regulated by its phosphorylated cofactors Hpr and Crh, and its phosphorylation is done by HprK. As such, expression of cellulase cannot happen in the presence of glucose in media. In our study, three mutations were found in the CcpA gene of the mutant compared to the wild type, but since these mutations did not affect the amino acid sequence, the function was not affected. Other studies reported that RL-P37 enhances its cellulase and endoglucanase activity by two-fold [39]. Cre1, which is a CCR protein of *T.ressei*, was found to be truncated in the hyper producer of cellulase in *T. reesei* Rut-C30 and demonstrated that Cre1 regulates the cellulase expression [40]. The disruption of *CreA* gene enhanced the production of cellulase and hemicellulase in *A.cellulolyticus* [41].

In *Bacillus subtilis,* CCR mechanism is regulated by the HprK catalyzed phosphorylation of Hpr (ser-46). Maximum levels of Hpr are phosphorylated on strongly repressing substrate, and minimum levels of Hpr phosphorylation are observed on weakly repressing substrates. Hence, the strength of the CCR is dependent upon the activity of the HprK/P –dependent de (phosphorylation) of Hpr. Consequently, low Hpr kinase activity limits CCR by weakly repressing sources [42]. In our study, three unique mutations occurred in the mutant without changing the amino acid sequence, implying no loss of function of HprK gene compared to its wild type (VS15).

There are several genes that can lead to inhibition of phospholipid synthesis like gpsA (Cronan et al. 1975 [43]; Loewy 1990 [44], MORBIDONI et al. 1995 [45]). The inhibition of phosphoplipid synthesis can cause leaky surface allowing the enhanced transport of cellulases to the exterior. There could be inhibition of peptidoglycan synthesis without affecting macromolecular synthesis or growth as was noted in *E. coli* (Rodionov et al. 1996 [46]).

Also, there are mutations found in penicillin binding proteins(pbpA, pbpB) with role in cell wall synthesis and elongation( Murray et al. 1997 [47]). The affect on peptidoglycan synthesis can cause the development of a leaky mutant. Cell wall endo-peptidases like YkfC (Xu et al. 2010 [48]), cwlO (Hashimotyo et al. 2012 [49]; Hashimotyo et al. 2018 [50]) can create hurdles in cell wall synthesis. 

There were mutations in spore coat components of cotT, cotB, spoIIQ or yeeK. Unfavourable conditions like starvation may induce sporulation. Lack of ready substrates like glucose may favour sporulation. However, in order to utilise and grow on alternative substrates like cellulose, the host inactivated sporulation genes. Other surface components included membrane protein, bacteriophage SPP1 receptor(yueB). However, their role cannot be related to cellulase activity.

There are mutations to be found in genes related to carbohydrate metabolism like endo-1,4-beta-xylanase(xylanaseD,xynD),beta-galacturonidase(yesZ), alpha;-galacturonidase (lplD), chitosanase(csn). Probably, these genes or pathways compete for the intermediates or metabolites of the pathway involving cellulases. Possibly the metabolites of cellulase activity can act as inducers for these genes or operons, and during selective pressure of the genome shuffling, these genes were silenced.

Genes of regulatory origin have also been identified as mutants. These include rsbX(serine phosphatase), gutR (transcriptional regulator of the glucitol operon), yhcZ(two-component response regulator ,YhcY), fatR(transcriptional regulator for cypB), yobD((transcriptional regulator; phage-related; Xre family)).

Also deletions/insertions were found in other genes hemN(coproporphyrinogen III oxidase), pKSM(polyketide synthase), rseP (inner membrane zinc metalloprotease required for the extracytoplasmic stress response mediated by sigma€, YaeL), rok (repressor of comK), plipastatin synthetase(PpsC), swrAA (motility and swarming), SwrC(surfactin self-resistance) tuaA(teichuronic acid synthesis). 

Rok is a repressor of the transcriptional activator ComK and is therefore an important regulator of competence in Bacillus subtilis and is also proved to be a suppressor of membrane bound and secreted proteins. HtpX is an integral cytoplasmic membrane metalloprotease facillitating growth especially at high temperatures is repressed by rok(Lin et al. 2018 [51]). In the event of mutation in rok, the host is able to grow well at high temperatures. Mutant of rok displayed altered colony morphology (Kovacs et al. 2011 [52]).

Sigma (E) is required for expression of phospholipids, membrane proteins etc during stress conditions. Inactivation of rseP may cause lack of activity of sigma(E), affecting phospholipid synthesis or membrane synthesis resulting in leaky mutants. 

The tuaA gene is involved in the incorporation of teichuronic acid into cell walls of B.subtilis (Qi et al. 1998 [53]). The mutations in tuaA may cause decrease in level of teichuronic acid in cell wall, possibly leading to a leaky mutant. 

The fatR protein represses the expression of the fatR-cyp102A3 operon involved in fatty acid detoxification.Unsaturated fatty acids have the capacity to interact with FatR and prevent repression (Gustafsson et al. 2001 [54]). There is accumulation of fatty acids during inhibition of phospholipid synthesis (like during inactivation of gpsA). Mutation in fatR prevents its repression activity.This may cause the expression of fatR-cyp102A3 operon facilitatiing fatty acid detoxification or surviving fatty acid accumulation.

The gene YhcZ forms part of two component regulatory system (YfcZ-YfcY) and promotes glucsoe and carbon source utilisation (Jia et al. 2018). Mutations in the yhcZ may lead to conditions of carbon starvation,activation of the carbon catabolite repressor pathways, that cause induction of cellulase activity.

*Bacillus subtilis* can utilize glucitol as a sole carbon source via the gut operon (Watanabe et al. 2003 [55]). The gene gutR can act as regulator of gut operon. Mutantion of gutR, could cause the host to utilise the xylitol(CHALUMEAU et al. 1978 [56]). Probably, it may also help the host in utilisation of other alternative substrates like cellulose.

Sigma(B), stress response sigma factor of *Bacillus subtilis*, is induced during environmental stress or at low cellular ATP levels.The gene, rsbX is a negative regulator of of sigma(B) (Smirnova et al. 1998 [57]; Teh et al. 2015 [58]). Mutation in rsbX, continues the stress response and possible utilisation of alternative substrates pathways (like cellulases) to replenish ATP levels.

The molecular membrane protein chaperon, prsA is also mutated. This chaperon has been implicated in increased secretion of amylase(Quesada-Ganuza et al. 2019 [59]), lipases (Ma et al. 2018 [60]) and several other proteins(Kakeshita et al. 2011 [61]). However, the role of the frameshift mutation in prsA resulting in increased cellulase activity cannot be explained. Similar is the case with mutation in another chaperon, GroeL. 

Mutation in SwrAA involved in motility and swarming and gamma- poly glutamic acid synthesis was observed(Osera et al. 2009 [62]) . The gene (SwrC) conferring resistance to surfactin produced by the host was also mutated. Also, there were mutations to be found in a polyketide synthesis operon,and the plipastatin production operon. The gene hemN facilitating heme synthesis in anerobic conditions(Hippler et al. 1997 [63]) was mutated. The gene(pucF; allantoate amidohydrolase) involved in purine degradation as nitrogen source(Schultz et al. 2001 [64]) was mutated. However, their role in cellulase activity cannot be predicted.

## 4. Materials and Methods

### 4.1. Material

The Substrates (Caroxymethylcellulose (CMC) and Whatman No.1. filter paper, (GE healthcare, Buckinghamshire, UK) and chemicals (3, 5-dinitrosalicyclic acid (DNS), and NTG *N*-Methyl-*N*′ nitro-*N*-nitrosoguanidine (NTG)) were purchased from Himedia Laboratories Private Limited, Mumbai, India , where Cellobiose from Sigma Aldrich, St. Louis, USA.

All other reagents are of extra pure grade was obtained from Merck & co., Bengaluru, India. 

Cotton gin waste was procured from Kallam Agro Products & Oils Pvt Ltd, Guntur, India.

### 4.2. Microorganism and Culture Conditions

*Bacillus subtilis* VS15 strain (GenBank: KT210118.1) previously isolated from decomposing logs is used as the host organism for genome shuffling [65]. The Cellulase producing bacteria were isolated and cultivated in CMC media [66], supplemented with 1% of NH_4_H_2_PO_4_. 100 µL of soil sample inoculum from the serial dilution was spread on CMC plates and incubated at 37 °C for 16–18 h. Colonies were screened based on the zone of clearance using Gram’s iodine and further confirmed by quantitative analysis by determining the reducing sugars liberated from each isolate through the dinitrosalicylic acid (DNS) method [67]. The strain was maintained on carboxy methyl cellulose (CMC) agar slants and was preserved in a CMC broth with 50% (*w*/*v*) glycerol at −80 °C.

### 4.3. Culture Conditions

A loop full of overnight culture was inoculated in a 250 mL flask containing 8% wheat bran substrate medium [68] supplemented with 1% of NH_4_H_2_PO_4_, and the flask culture was kept in an orbital shaker at 37 °C, 150rpm for 54 h. After incubation, a 1% rate of seed culture was inoculated into 25 mL of production medium in a 250 mL Erlenmeyer flask and incubated with the same seed culture conditions. 

### 4.4. Mutagenesis

Cells of VS15 were mutagenized with NTG (2–5 µg grains), EMS (0.620–1.48 mM, and UV (1–10 min) at various conditions, and optimal conditions were evaluated. In method 1, cells at OD_600_ of 1.0 were suspended in 0.931 mM of EMS concentration for an hour. In method 2, cells were treated with 2 µg grains of NTG for 30 min. In method 3, cells were irradiated with UV (Philips 30W G30 TB) with a wavelength of 360 nm at a distance of 35 cm for 3 min. In method 4, treatment was done with a combination of 2 µg grains of NTG for 30 min, and UV irradiation for 3 min. In method 5, treatment was done with a combination of 0.931 mM of EMS concentration for an hour and UV irradiation was conducted for 3 min. Then, the treated cells were pooled and spread on the CMC media plated, which was incubated at 37 °C for 16 h. Mutants were screened based on the fastest formation of clear halos around the colonies, and further confirmation was done by the DNS method.

### 4.5. Genome Shuffling

Protoplast fusion was performed according to the method described by reference [7]. Selected mutants of VS15 strains were cultured in a CMC broth with 1% glycine at 50 °C for 16 h. The resultant cells were collected by centrifugation at 6000× *g* for 10 min and washed three times with protoplast buffer and incubated with a mixture of lysozyme (10 mg/mL) mutanolysin (5 mg/mL), and sucrose (0.5 M) at 37 °C for 60 min. Sucrose was added as an osmotic stabilizer to the cell suspension according to the method from reference [69]. Protoplast suspension was equally divided into two parts. One part was irradiated with UV for 30 min, and the other part was treated with heat at 60 °C for 2 h [70]. Inactivated protoplasts of the two different methods were pooled after centrifugation and resuspended in a HEPES buffer. PEG 6000 was used according to the method from reference [71]. Various concentrations (70%, 80%, and 90%) of PEG were examined and 70% of PEG 6000 with 20 mM CaCl_2_ was chosen for efficient protoplast fusion.

These protoplasts were fused under nine volumes of PEG 6000 and one volume of protoplast buffer and incubated. During the incubation later at 37 °C for 30 min, the fused protoplast suspension was centrifuged, washed, and resuspended in 1 mL of protoplast buffer. Further, the suspension was diluted three fold, plated on CMC media plates, and incubated at 50 °C for 16 h. Colonies with clear halos were taken for the next round of genome shuffling. The probability of protoplast formation was calculated according to the method from reference [11].

### 4.6. Effect of Various Carbon Sources of Enzymatic Yield of Wild and Shuffled Strain

Three different carbon sources such as Carboxymethylcellulose (CMC), Cellobiose, and Filter paper were used to check the effect of cellulase production. Here an overnight culture of wild (VS15) and mutant (Gd7) cultures was inoculated separately in wheat bran broth at 8% (*v*/*v*) and incubated for 54 h at 37 °C with 150 rpm in an orbital shaker. After incubation, the samples were centrifuged (Thermo scientific st16r) at 10,000 rpm for 5 min at 4 °C. The supernatant was collected to carry out CMCase, Filter paper, and Cellobiose assays to estimate the protein titer according to the method from reference [72]. All the assays were carried out in triplicates. One unit of cellulase is defined as the amount of enzyme that liberates reducing sugar at the rate of 1 µmol/min under the assay conditions [73]. 

In the case of CMCase assay, 2% of carboxymethyl cellulose (CMC) in Na citrate buffer (0.05 M, pH 4.8, 0.5 mL) was added to 0.5 mL of properly diluted enzyme, and it was allowed to incubate at 50 °C for 30 min. After the incubation period, the reaction was stopped by adding 3 mL of 3, 5-Dinitro-salicylic-acid(DNS) to all the tubes. The reaction tubes were boiled for 5 min in a vigorously boiling water bath. The absorbance was read at 540 nm using a UV-Visible spectrophotometer (HALO DB20 UV- Visible double beam spectrophotometer).

Filter paper activity was estimated using Whatman No. 1 filter paper 1.0 × 6.0 cm (≈50 cm) as a substrate. The activity was carried out in a reaction mixture consisting of 1.0 mL of 0.05 M Na citrate (pH 4.8), 0.5 ml of properly diluted enzyme solution, and 1 filter paper strip. This mixture was incubated at 50 °C for 60 min. After the incubation period, the reaction was stopped by adding 3 mL of 3 and 5-Dinitro-salicylic-acid (DNS) to all the tubes. The reaction tubes were boiled for 5 min in a vigorously boiling water bath. The absorbance was read at 540 nm using a UV-Visible spectrophotometer (HALO DB20 UV-Visible double beam spectrophotometer).

For the cellobiase assay, 15.0 mM cellobiose in 0.05 M citrate buffer (pH 4.8) of 1.0 mL was added to 1.0 mL of properly diluted enzyme mix and incubated at 50 °C for 30 min, and the reaction was terminated by boiling for 5 min. The glucose released was estimated using the glucose oxidase method.

### 4.7. Utilization of Cotton Gin Waste (CGW) as a Substrate

Application of cellulase extends to several industries like the paper, pulp textile, bio fuel, food, beverages, and detergent industries; bio fuel industries have great demand in order to help assess the utilization of cotton gin waste as a substrate. The raw material was treated with H_2_SO_4_, while approximately 5 g of CGW was weighed and suspended in the H_2_SO_4_ solution (1%, 3%, 5%, and 7%) in the ratio of 1:10 (*w*/*v*) and the mixture was autoclaved at 121 °C for 15 min [74,75]. After cooling the hydrolysate was pressed through the cheese cloth and used as a substrate. The properly diluted supernatant (0.5 mL) of wild and shuffled strains was incubated with 0.5 mL of pre-treated cotton gin waste at 50 °C for 30 min, and the liberated reducing sugars was estimated by using Millers method.

### 4.8. Short Evolution of Ethanol Production Acidified Potassium Dichromate

Short evolution of ethanol production was conducted by acidified potassium dichromate method. Both VS15 and Gd7 were grown in a 8% wheat bran medium at 37 °C and 150 rpm for 54 h. After incubation, the culture broths were inoculated by 1% *saccharomyces cerevisae* with the addition of 0.04% KH_2_PO_4_, 0.005% CaCO_3_, 0.002% MgSO_4_, and 0.001%NaCl, and incubated for five days [76] and the amount of ethanol produced by fermentation was estimated by the potassium dichromate method [77].

### 4.9. NGS Sequencing and Data Processing

The strains were grown in the cell culture, and the total genomic DNA was extracted from purified two *Bacillus subtilis* strains (VS15 and Gd7) and converted to sequencing libraries using the TrueSeq DNA nano kits (Illumina, San Diego, USA. Libraries were normalized and pooled before sequencing on an Illumina HiSeq2500 with 2 × 101 paired-end reads for Gd7. Whereas for VS15, sequencing on an Illumina MiSeq with 2 × 300 paired-end reads. The NGS sequencing of *Bacillus subtilis* VS15 (wild) was carried at Latrobe University (Australia) and the *Bacillus subtilis* Gd7 (mutant) sequencing was done at Macrogen (Seoul, South Korea).

#### 4.9.1. Assembly and Annotation

There were two samples used from the strain wild type (VS15) and mutant (Gd7). The A5-miseq pipeline (6) was used to perform read trimming and correction, contigs assembly, crude scaffolding, misassembly correction, and final scaffolding. All assemblies were annotated to evaluate gene set completeness using online RAST (Rapid Annotations using Subsystems Technology) Server RAST identifies protein-encoding, rRNA, and tRNA genes (Figure S1a,b), assigns functions to the genes, and predicts which subsystems are represented in the genome of wild type (VS15) and mutant (Gd7) (Figure S2a,b).

#### 4.9.2. SNP Calling

Raw reads from both the samples i.e., VS15 and Gd7 were separately mapped to *Bacillus subtilis subsp. subtilis str. 168* genome using bowtie2 aligner [78]. The overall alignment rate for VS15 was 91.30% whereas for Gd7 was 90.67%. The alignment SAM file was then converted into the BAM format using SAMTools [79] to further view its utility. We then used SAMTools mpileup and VarScan.v2.3.9 [80] with default parameters for variation calling, and used SnpEff [81] for variation effect prediction. For effect prediction, we used *Bacillus subtilis subsp. subtilis str. 168* annotation downloaded by SNPEff download utility from ensemble. We also used DELLY [82] with default parameters for the prediction of structural variations (SVs) in both the samples wild VS15 (Table S1) and mutant Gd7 (Table S2).

#### 4.9.3. Repeat Analysis

Various repeat finding tools have been used to identify repetitive elements from both assemblies (VS15 and Gd7). FullSSRversion-1.1 (https://sourceforge.net/projects/fullssr/) a standalone perl script with default parameters has been used for the prediction of simple sequence repeats (SSRs). We have used EMBOSS version-6.6.0package for the inverted, tandem, and palindromic repeat sequences in both VS15 and Gd7 assembly with default parameters. Direct repeat sequences analysis has been done using Red: an open source tool for repeat prediction with its default parameters.

### 4.10. Specific Gene Set Analysis for SNP

We have selected few genes and pathways that are significantly involved in cellulase production and cellulase activity. From this gene set, most of the genes have been affected by the SNPs. The variant position for selected gene set in both the samples i.e., VS15 and Gd7 is provided in File S1a. It also includes the information about gene boundaries, SNP positions, nucleotide base present in the reference, as well as the altered nucleotide base at the same position.

## 5. Conclusions

Genome shuffling was applied to bacterial strain in which the cellulase activity of *Bacillus subtilis* VS15 was enhanced by 167%. *Bacillus subtilis* VS15 and Gd7 show enzyme activity at extreme temperatures (50 °C), which is much more beneficial for industrial processes. The whole genomes of the wild and shuffled strain were obtained by NGS and compared. There were differences in the genome sequences that demonstrated the effect of genome shuffling. A set of genes were analyzed to infer the cause for enhanced cellulase production. It can be infered that the cause of enhanced cellulase production in the shuffled strain is due to the interaction of multiple mutations of multiple genes. Production of ethanol with the help of *Saccharomyces cerevisiae* indicated that the cellulase activity can be used in the production of biofuel from cellulosic residues. This is the first report comparing the genomes of wild and shuffled strains. 

## Figures and Tables

**Table 1:** Cellulase production of mutant strains obtained by combination of mutagenesis (EMS+UV) and (NTG+UV) compared to wild strain VS15.

Mutagenesis	Strains	IU/mL
EMS+UV	EUA9	4.56
	EUB8	5.93
	EUD6	5.36
NTG+UV	NUA7	5.15
	NUB2	4.92
	NUD8	5.49
Control	VS15	3.77

**Table 2:** Cellulase activities of the *Bacillus subtilis* VS15 and its shuffled strains incubated with 8% of Wheat bran as a carbon source for 54 h.

Activity	VS15	Specific Activity	Gb2	Specific Activity	Gc8	Specific Activity	Gd7	Specific Activity
CMCase (IU/mL)	3.8 ± 0.01	4.±0.04	8.7 ± 0.05	5.8 ± 0.04	9.5 ± 0.03	6.25 ± 0.01	10.02 ± 0.03	5.7 ± 0.01
FPase (IU/mL)	2.4 ± 0.02	2.52 ±0.01	3.8 ± 0.02	2.5 ± 0.03	4.2 ± 0.02	2.9 ± 0.01	4.5 ± 0.02	2.6 ± 0.03
Cellobiose (IU/mL)	12 ± 0.01	12.6 ±0.03	18.9 ± 0.01	12.7 ± 0.04	19.2 ± 0.01	12.6 ± 0.02	20.2 ± 0.02	12 ± 0.05
Protein Conc. (mg/mL)	0.95 ± 0.04		1.5 ± 0.03		1.52 ± 0.01		1.75 ± 0.01	

**Table 3:** *Bacillus subtilis sp.* genome assembly statistics.

Statistic	VS15	Gd7
Total number of Contigs	67	63
Total number of Scaffolds	67	63
Total number of Bases	4,163,202	4,146,024
Average Scaffold Length	62.13734 Kb	65.8099
Median Scaffold Length	1572 bp	403 bp
N50	0.293644 Mb	1.023553 Mb
N90	60.193 Kb	224.741 Kb
GC Content	43.71%	43.65%
SNP Rate	9.79/4215.6 Kb	11.04/4215.6 Kb

**Table 4:** *Bacillus subtilis sp.* genome annotation.

Statistic	VS15	Gd7
Number of genes predicted	4426	4375
Number of scaffolds containing predicted genes	67	63
Number of Subsystems	482	477
Number of protein coding genes	4316	4278
Number of non-protein coding genes	110	97
Number of characterized protein	3317	3287
Number of hypothetical/putative protein	1038	1017
Number of rRNA genes	84	79
Number of tRNA genes	26	18
Number of proteins with Pathway Annotation	955	950

**Table 5:** SNP across specific gene set in VS15 and Gd7 sample.

Sr. No.	Gene Name	Annotation	SNPs in VS15	SNPs In Gd7
1	*bglA*	aryl-6-phospho-beta-glucosidase	24	21
2	*bglC*	aryl-phospho-beta-d-glucosidase	9	9
3	*bglH*	aryl-phospho-beta-d-glucosidase	19	20
4	*eglS*	endo-1,4-beta-glucanase	30	31
5	*licT*	transcriptional antiterminator (BglG family)	1	1
6	*hprK*	Hpr kinase/phosphorylase	8	11
7	*CcpA*	transcriptional regulator involved in carbon catabolite control	6	9
8	*yxaL*	serine/threonine protein kinase	11	14
		Grand Total	108	115

**Table 6:** InDels across the specific gene set in a VS15 sample.

Sr. No.	Gene Name	Gene Start	Gene End	InDel Position	Reference Bases	InDels	Altered Base Composition	Upstream Distance of InDel From Gene (Kb)
1.	eglS	1940625	1942124	1938626	A	+TGTT	ATGTT	−1.999
2.	bglC	370259	371692	365852	G	+A	GA	−4.407
3.	licT	4012866	4013699	4007939	G	+T	GT	−4.927

**Table 7:** InDels within coding region in VS15 sample.

Gene Name	Gene Start	Gene End	InDel Position	Reference Base	Indel
csn	2747984	2748817	2748774	G	-CTGCTTTTTTCCAAAAAT
cwlO	3574363	3575784	3574858	A	-GAAGAATTGTCT
tuaA	3658259	3658408	3658405	C	+CCAGCCGCTCAATCCTGGTTTAACAG
spoIIQ	3759702	3760553	3759864	A	+GCTTTTTCTTCAGCAGCTGCT

**Table 8:** InDels across specific gene set in a Gd7 sample.

Sr. No.	Gene Name	Gene Start	Gene End	InDel Position	Reference Bases	InDels	Altered Base Composition	Upstream Distance of InDel From Gene (Kb)
1.	bglC	370259	371692	369225	T	+G	TG	−1.034
2.	licT	4012866	4013699	4011724	T	+GAG	TGAG	−1.142
3.	licT	4012866	4013699	4011712	T	+ACA	TACA	−1.154
4.	eglS	1940625	1942124	1938628	G	+T	GT	−1.997
5.	eglS	1940625	1942124	1938626	A	+TGTT	ATGTT	−1.999
6.	bglC	370259	371692	365852	G	+A	GA	−4.407
7.	licT	4012866	4013699	4007939	G	+T	GT	−4.927

**Table 9:** InDels across specific gene set in a Gd7 sample.

Gene Name	Gene Start	Gene End	InDel Position	Reference Base	InDel
rsbX	523650	524249	524249	A	+G
groEL	650234	651868	651817	C	-GCA
gutR	664775	667264	665353	G	+T
swrC	732916	736113	736111	T	+AA
yeeK	753265	753702	753407	A	+TGG
yesZ	774799	776790	776726	A	-G
lplD	782958	784298	784292	A	+CGTTG
yhcZ	1009804	1010448	1010143	C	+TAA
prsA	1070364	1071242	1070495	C	+AT
prsA	1070364	1071242	1070658	G	+A
cotT	1280626	1280949	1280748	G	+ATA
cotT	1280626	1280949	1280755	C	+G
ykfC	1367941	1368831	1368350	T	-G
rok	1493787	1494362	1494344	A	+GAATCAGCT
rok	1493787	1494362	1494351	G	+CTGAAT
rok	1493787	1494362	1494352	C	+TG
pbpB	1581947	1584097	1582463	G	-GA
rseP	1724029	1725297	1725079	C	-A
pksM	1821553	1834341	1825732	C	+T
xynD	1944113	1945654	1944323	T	+GA
ppsC	1974881	1982548	1980780	C	-A
ppsC	1974881	1982548	1980783	T	+G
yobD	2056278	2056616	2056405	T	-G
gpsA	2389151	2390188	2389986	G	-C
pbpA	2581771	2583921	2581907	A	-T
pbpA	2581771	2583921	2581910	G	-A
pbpA	2581771	2583921	2581919	T	+A
hemN	2629718	2630857	2630855	C	+A
fatR	2777070	2777654	2777091	C	-T
yueB	3266687	3269917	3268994	T	+AAAC
pucF	3342433	3343671	3343173	C	+A
pucF	3342433	3343671	3343175	G	-T
swrAA	3621931	3622047	3621938	A	-T
tuaA	3658259	3658408	3658402	G	+GTTT
cotB	3714739	3715881	3714900	A	+ACTCT
pdp	4049009	4050310	4049924	G	-C
fbp	4128029	4130044	4130044	A	-G

## Supplementary Materials

Supplementary materials can be found at https://www.mdpi.com/1422-0067/21/4/1299/s1.
