# Supplementary material for: Comparative Analysis of Structural Variations Due to Genome Shuffling of Bacillus Subtilis VS15 for Improved Cellulase Production
Source: Int J Mol Sci. 2020 Feb 14;21(4):1299. doi: 10.3390/ijms21041299 (PMC7072954; doi:10.3390/ijms21041299)
Supplement: Supplementary file 1 [file ijms-21-01299-s001.zip › supplementary/Supplementary_file_s3.pdf]

List of CAzymes from *Bacillus subtilis subsp. Subtilis* 168

Protein Name	Family	Reference Accession
BSU15220 (MurG)	GT28	CAB13395.1
α-1,6-glucosidase (MalL;YvdL;BSU34560)	GH13	CAB15461.1
α-amylase (AmyE;AmyA;BSU03040)	GH13,CBM26	BAA08938.1
α-galactosidase (MelA;BSU30300)	GH4	AAC00383.1
α-galacturonidase (LplD;BSU07130)	GH4	CAB12532.1
α-L-arabinofuranosidase 1 (AbfA;BSU28720)	GH51	CAA99595.1
β-1,4-xylosidase (BsX;XynB;YnaK;BSU17580)	GH43	AAB41091.1
β-galactosidase (LacA;YvfN;BSU34130)	GH42	CAB15418.1
β-galactosidase (YesZ;BSU07080)	GH42	CAB12527.1
β-mannanase (YdhT;BSU05880)	GH26	BAA19712.1
β-N-acetylglucosaminidase (NagZ;YbbD;YzbA;BSU01660)	GH3	AAA64351.1
6-P-α-glucosidase (GlvA;Glv-1;GlgG;BSU08180)	GH4	BAA09103.1
6-P-β-glucosidase (BglA;BSU40110)	GH1	AAA22660.1
6-P-β-glucosidase (CelF;CelD;LicH;BSU38560)	GH4	CAA90288.1
6-P-β-glucosidase (YckE;BglC;BSU03410)	GH1	CAB12135.1
α-L-arabinofuranosidase 2 (AbfA2;Abf2;Xsa;Asd;BSU28510)	GH51	CAA99576.1

6-P-β-glucosidase (YdhP;BSU05840)	GH1	BAA19708.1
6-P-β-glucosidase 1 (BglH;N17D;BSU39260)	GH1	CAB15962.2
acetyl xylan esterase (YesT;BSU07020)	CE12	CAB12521.1
acetyl xylooligosaccharide esterase /cephalosporin-C deacetylase (Cah;BSU03180)	CE7	CAB12112.1
arabinoxylan α-L-1,3-arabinofuranohydrolase (XynD;BsAXH-m23;BSU18160)	GH43,CBM6	CAB13699.1
autolysin / endopeptidase (CwlS;YojL;BSU19410)	CBM50,CBM50,CBM50,CBM50	CAB13833.1
bifunctional muramidase DD-endopeptidase (CwlP;YomI;BSU21350)	GH23	CAB14053.2
bifunctional muramidase soluble-lytic transglycosylase (CwlQ;YjbJ;BSU11570)	GH23	AIY92440.1
bifunctional N-acetylmuramidase / D-,L-endopeptidase (CwlT;YddH;BSU04970)	GH23	CAB12304.1
branching-enzyme (GlgB;GBE;BSU30980)	CBM48,GH13	AAC00214.1
BSU00160 (YaaH)	CBM50,CBM50,GH18	CAB11792.1
BSU01570 (YbaN;YbxG;PdaB)	CE4	BAA10997.1
BSU02840 (YcdG)	GH13	BAA22245.1
BSU04300 (YdaM)	GT2	BAA19267.1
BSU05710 (YdhD)	CBM50,CBM50,GH18	CAB12390.1
BSU05720 (YdhE)	GT1	BAA19696.1
BSU07090 (YetA)	PL26	CAB12528.2
BSU07290 (YfnF)	GTnc	CAB12548.1
BSU07300 (YfnE)	GT2	CAB12549.1
BSU07310 (YfnD)	GTnc	CAB12550.1
BSU08600 (YfhN;CsbB)	GT2	AAB38429.1
BSU09370 (LytF)	CBM50,CBM50,CBM50,CBM50,CBM50	CAB12776.2
BSU09420 (LytE)	CBM50,CBM50,CBM50	CAB12781.2
BSU09660 (YheN)	CE4	CAA74511.1
BSU12100 (YjeA)	CE4	AAC46306.1
BSU12220 (YjiC)	GT1	AAC46318.1
BSU12890 (YkcC)	GT2	CAA05569.1
BSU13350 (YkoN)	GT28	CAB13192.1
BSU13390 (YkoT)	GT2	CAB13196.1
BSU14040 (YkuD)	CBM50	CAA10867.1
BSU16700 (YlxY;YmxI)	CE4	CAB13543.1
BSU18650 (PelB)	PL1	CAB13757.1
BSU19130 (YocA)	GH23	CAB13805.1
BSU19210 (YocH)	CBM50,CBM50	CAB13813.1
BSU19420 (YojK)	GT1	CAB13834.2
BSU19760 (CgeD;CgeBB)	GT2	AAA87718.1
BSU22470 (YpjG)	CE14	CAB14163.2
BSU24910 (YqgM)	GT4	CAB14421.1
BSU30880 (YtcC)	GT4	AAC00365.1
BSU30910 (YtxN;CotSA)	GT4	AAC00219.1

BSU31290 (YugT)	GH13	CAB07926.1
BSU34020 (YvbX)	GH18	CAB15407.1
BSU34280 (YveT)	GT2	CAA96477.1
BSU34300 (YveR)	GT2	CAA96475.1
BSU34320 (YveP)	GT4	CAA96473.1
BSU34330 (EpsE)	GT2	CAB15438.1
BSU34340 (YveN)	GT4	CAA96471.1
BSU34570 (YvdK)	GH65	CAB08040.1
BSU35540 (TuaH)	GT4	CAB15571.1
BSU35550 (YvhG;TuaG)	GT2	CAB15572.1
BSU35590 (YvhC;TuaC)	GT4	AAB94864.1
BSU36460 (YwoF)	PL9	CAB15663.1
BSU37510 (YwhE)	GT51	CAB02515.1
BSU37980 (YwdF)	GT2	CAB15824.1
BSU38430 (GspA;YwaG) (possible fragment)	GT8	CAA51568.1
BSU38800 (YxkH;YxkH)	CE4	CAB15906.1
BSU39120 (YxiM;Ss8D)	CE12	CAB15948.1
chitosanase (Csn;BSU26890)	GH46	CAB14630.1
endo-α-1,5-L-arabinanase (Abn2;YxiA;J3A;BSU39330)	GH43	CAB15969.2
endo-α-1,5-L-arabinanase (AbnA;BSU28810)	GH43	CAA99586.1
endo-β-1,4-glucanase (EglS;BglC;Gld;BSU18130;BsCel5;BsCel5A)	GH5,CBM3	CAA82317.1
endo-β-N-acetylglucosamidase (LytD;CwlG;BSU35780)	GH73	AAA67857.1
endo-levanase (YveB;LevB;BSU34460)	GH32	CAB08014.1
endo-rhamnogalacturonan lyase (YesW;BSU07050)	PL11	CAB12524.1
exo-unsaturated rhamnogalacturonan lyase (YesX;BSU07060)	PL11	CAB12525.1
expansin (Exlx1;BsEXLX1;YoaJ;ExpA;BSU18630)	CBM63	AAB84448.1
galactanase (GalA;YvfO;BSU34120)	GH53	CAB08009.1
glucuronoxylan xylanohydrolase (feraxanase) / xylanase C (XynC;YnfF;BSU18150)	GH30	CAA97612.1
glycogen phosphorylase (GlgP;BSU30940)	GT35	AAC00218.1
glycogen synthase (GlgA;BSU30950)	GT5	AAC00217.1
GP2222_15230 (Ykud_1)	CBM50	AQR81381.1
invertase (SacA;BSU38040;IPA-50D)	GH32	AAA22723.1
levanase (SacC;BSU27030)	GH32,CBM66	CAB14645.1
levansucrase (SacB;BSU34450)	GH68	CAA26513.1
lichenase (BglS;LicS;Bgl;BSU39070)	GH16	CAA86922.1
maltogenic α-amylase / neopullulanase (YvdF;BSU34620)	CBM34,GH13	CAB08035.1
N-acetylglucosamine 6-phosphate deacetylase (NagA;BSU35010)	CE9	CAB15506.1
pectate lyase (Pel;Pel168;BSU07560)	PL1	CAB12585.1
pectate lyase C (PelC;YvpA;BSU34950)	PL3	CAB15500.1

pectin acetylesterase (YesY;BsPAE;BSU07070)	CE12	CAB12526.1
penicillin-binding protein 1A (PbpF;PonA;Pbp2c;BSU10110)	GT51	CAA74517.1
penicillin-binding protein 1A / 1B (PonA;Pbp1A/B;BSU22320)	GT51	AAA64947.1
penicillin-binding protein 4(PbpD;BSU31490)	GT51	AIY95549.1
peptidoglycan hydrolase (LytG;YubE;BSU31120)	GH73	CAB15090.1
peptidoglycan N-acetylmuramic acid deacetylase (YfjS;PdaA;BSU07980)	CE4	CAB12627.1
processive 1,2-diacylglycerol-3-β-glucosyltransferase (UgtP;YpfP;BSU21920)	GT28	AAA96624.1
pullulanase type I / glycogen-debranching enzyme (AmyX;BSU29930)	CBM68,CBM48,GH13	AAC00283.1
QU35_02455	GH126	AIY91710.1
QU35_06945	CBM50	AIY92535.1
QU35_07085	GH23	AIY92563.1
QU35_07090	CBM50	AIY92564.1
QU35_07155	CBM50	AIY92577.1
QU35_07670	CBM50	AIY92675.1
QU35_07675	CBM50	AIY92676.1
QU35_07680 (fragment)	GH18	AIY92677.1
QU35_07685 (fragment)	GH18	AIY92678.1
QU35_12600	CBM50	AIY93621.1
QU35_14175	CBM50	AIY93925.1
QU35_14180	GH23	AIY93926.1
QU35_15145	CBM50	AIY94110.1
QU35_15290	CBM50	AIY94139.1
QU35_20460	GTnc	AIY95099.1
SpsA (BSU37910)	GT2	CAB15817.1
teichoic acid biosynthesis protein (GgaA;BSU35690)	GT2	AAA73512.1
teichoic acid biosynthesis protein (GgaB;BSU35680)	GT2	AAA73513.1
teichoic acid biosynthesis protein E (TagE;RodD;GtaA;GtaD;BSU35730; α-glucosyltransferase)	GT4	CAA33270.1
trehalose-6-phosphate hydrolase (TreA;BSU07810)	GH13	CAB12610.1
UDP-Glc: (sublancin 168) S-β-glucosyltransferase / sublancin S-glycosyltransferase (SunS;YolJ;BSU21450)	GT2	CAB14063.1
UDP-GlcNAc: L-malate α-N-acetylglucosaminyltransferase (YpjH;JojH;BshA;BSU22460)	GT4	AIY93568.1
UDP-ManNAc: α-GlcNAc-PP-C55 β-N-acetyl-mannosaminyltransferase (TagA;BSU3575)	GT26	AAA22844.1
unsaturated rhamnogalacturonyl hydrolase (BSU30120; YteR)	GH105	CAB14990.1
unsaturated rhamnogalacturonyl hydrolase (YesR;BSU07000)	GH105	CAB12519.1

xylanase A (XynA;BsxA;BsXynA;BSU18840)	GH11	**CAB13776.1**
