# Supplementary material for: Comparative Analysis of Structural Variations Due to Genome Shuffling of Bacillus Subtilis VS15 for Improved Cellulase Production
Source: Int J Mol Sci. 2020 Feb 14;21(4):1299. doi: 10.3390/ijms21041299 (PMC7072954; doi:10.3390/ijms21041299)
Supplement: Supplementary file 1 [file ijms-21-01299-s001.zip › supplementary/Supplementary_file_s4a.pdf]

- NCBI Home
- Sign in to NCBI
- Skip to Main Content
- Skip to Navigation
- About NCBI Accesskeys

U.S. National Library of Medicine

NCBI National Center for Biotechnology Information

-
- My NCBI
- Sign in to NCBI
- Register
- Sign Out

COBALT Constraint-based Multiple Alignment Tool

- Home
- Recent Results
- Help

Phylogenetic Tree Edit and Resubmit Back to Blast Results Download

Multiple Alignment Results - Protein Sequence - Cobalt RID 2UZ5BH39212 (2 seqs)

Graphical Overview

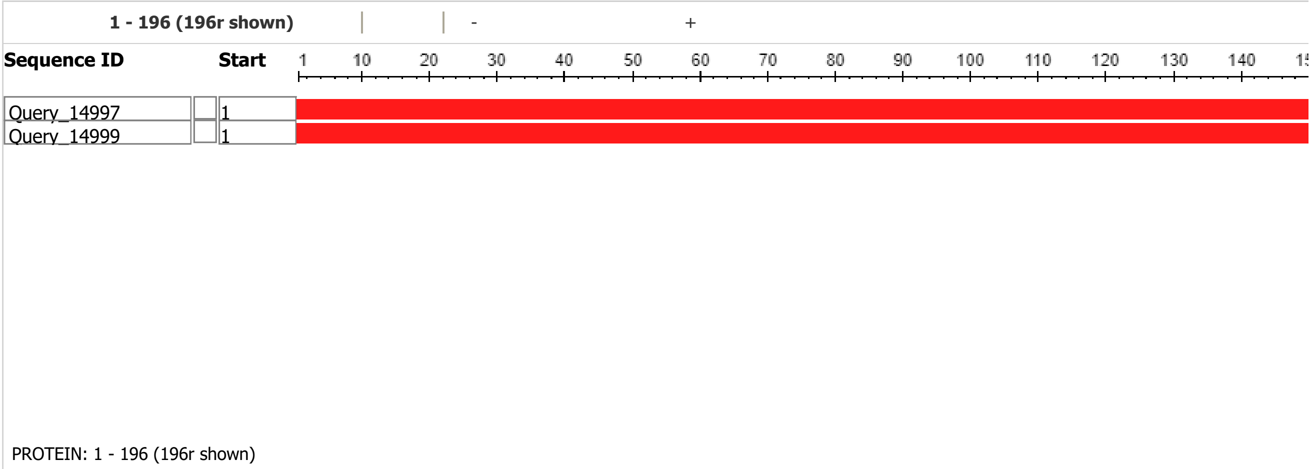

Descriptions

Select All Re-align Alignment parameters

Accession	Description	Links
lcl|Query_14997	unnamed protein product	
lcl|Query_14999	unnamed protein product	

Alignments

Select All Re-align Mouse over the sequence identifer for sequence title

View Format: Compact Conservation Setting: Identity

```
Query_14997  1    MLSASSSKYDMIMKASVSLFTERGFDATTIPMIAERAHVGTGTIYRYFDSKETLVNVLFQESIQRFTEKLKQDVSELPVR  80
Query_14999  1    MLSASSSKYDMIMKASVSLFTERGFDATTIPMIAERAHVGTGTIYRYFDSKETLVNVLFQESIQRFTEKLKQDVSELPVR  80
```

```
☑ Query_14997  81   EGFHHVFCCLVQFTKESDYALFFLETKKDAHYLNHTSKKMIENLTQMLDDYFNKGKAEGVIRSLPSNVLIAIVLGAFLKI  160
☑ Query_14999  81   EGFHHVFCCLVQFTKESDYALFFLETKKDAHYLNHTSKKMIENLTQMLDDYFNKGKAEGVIRSLPSNVLIAIVLGAFLKI  160

☑ Query_14997  161  YQLVQTGDIEMDTDLITELEQCCWDAISFIHHKN--  194
☑ Query_14999  161  YQLVQTGDIEMDTDLITELEQCCWDAI-KLHSSQK*  195
```

BLAST is a registered trademark of the National Library of Medicine.
Copyright | Disclaimer | Privacy | Accessibility | Contact | Send feedback
NCBI | NLM | NIH | DHHS
