# Supplementary material for: Comparative Analysis of Structural Variations Due to Genome Shuffling of Bacillus Subtilis VS15 for Improved Cellulase Production
Source: Int J Mol Sci. 2020 Feb 14;21(4):1299. doi: 10.3390/ijms21041299 (PMC7072954; doi:10.3390/ijms21041299)
Supplement: Supplementary file 1 [file ijms-21-01299-s001.zip › supplementary/Supplementary_file_s4b.pdf]

- NCBI Home
- Sign in to NCBI
- Skip to Main Content
- Skip to Navigation
- About NCBI Accesskeys

U.S. National Library of Medicine

NCBI National Center for Biotechnology Information

- 
- My NCBI
- Sign in to NCBI
- Register
- Sign Out


COBALT Constraint-based Multiple Alignment Tool


- Home
- Recent Results
- Help

Phylogenetic Tree Edit and Resubmit Back to Blast Results Download


# Multiple Alignment Results - Protein Sequence - Cobalt RID 32K6ZZTD21S (2 seqs)

## Graphical Overview

processing locations

No results available for the given RID: 32K6ZZTD21S

## Descriptions

☑ Select All Re-align Alignment parameters

| Accession | Description | Links |
|---|---|---|
| ☑ lcl\|Query_18663 | unnamed protein product | |
| ☑ lcl\|Query_18665 | unnamed protein product | |

## Alignments

☑ Select All Re-align Mouse over the sequence identifer for sequence title

View Format: Compact Conservation Setting: Identity

```
☑ Query_18663  1    MKKVTMLGAGSWGTALALVLTDNGNEVCVWAHRADLIHQINELHENKDYLPNVKLSTSIKGTTDMKEAVSDADVIIVAVP  80
☑ Query_18665  1    MKKVTMLGAGSWGTALALVLADNGNEVCVWAHRADLIHQINELHENKDYLPNVKLSTSIKGTTDMKTLFQTRISLSLLFQ  80
```

```
Query_18663  81   TKAIREVLRQAVPFITKKAVFVHVSKGIEPDSLLRISEIMEIELPSDVRKDIVVLSGPSHAEEVGLRHPTTVTASSKSMR  160
Query_18665  81   QKQFGKC*------------------------------------------------------------------------  88

Query_18663  161  AAEEVQDLFINHNFRVYTNPDIIGVEIGGALKNIIALAAGITDGLGYGDNAKAALITRGLAEIARLGTKMGGNPLTFSGL  240
Query_18665       --------------------------------------------------------------------------------

Query_18663  241  TGVGDLIVTCTSVHSRNWRAGNLLGKGYKLEDVLEEMGMVVEGVRTTKAAYQLSKKYDVKMPITEALHQVLFNGQKVETA  320
Query_18665       --------------------------------------------------------------------------------

Query_18663  321  VESLMARGKTHEMEDLVNTFENQVK*  346
Query_18665       --------------------------
```

BLAST is a registered trademark of the National Library of Medicine.
Copyright | Disclaimer | Privacy | Accessibility | Contact | Send feedback
NCBI | NLM | NIH | DHHS
