# Supplementary figures and images for: Comparative Analysis of Structural Variations Due to Genome Shuffling of Bacillus Subtilis VS15 for Improved Cellulase Production
Source: Int J Mol Sci. 2020 Feb 14;21(4):1299. doi: 10.3390/ijms21041299 (PMC7072954; doi:10.3390/ijms21041299)

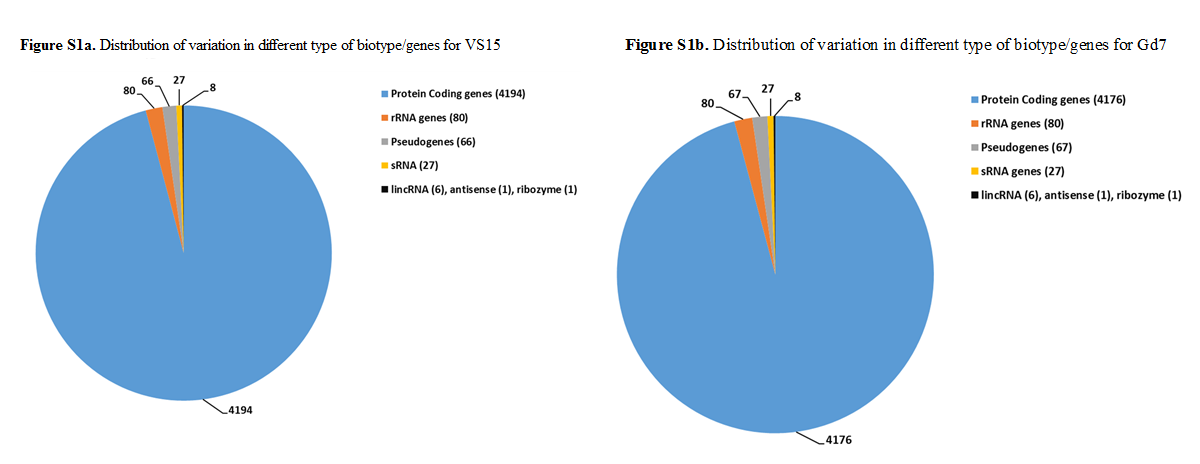

Supplement: Supplementary file 1 [file ijms-21-01299-s001.zip › supplementary/Supplementary_figures_S1A___S1b.tif]

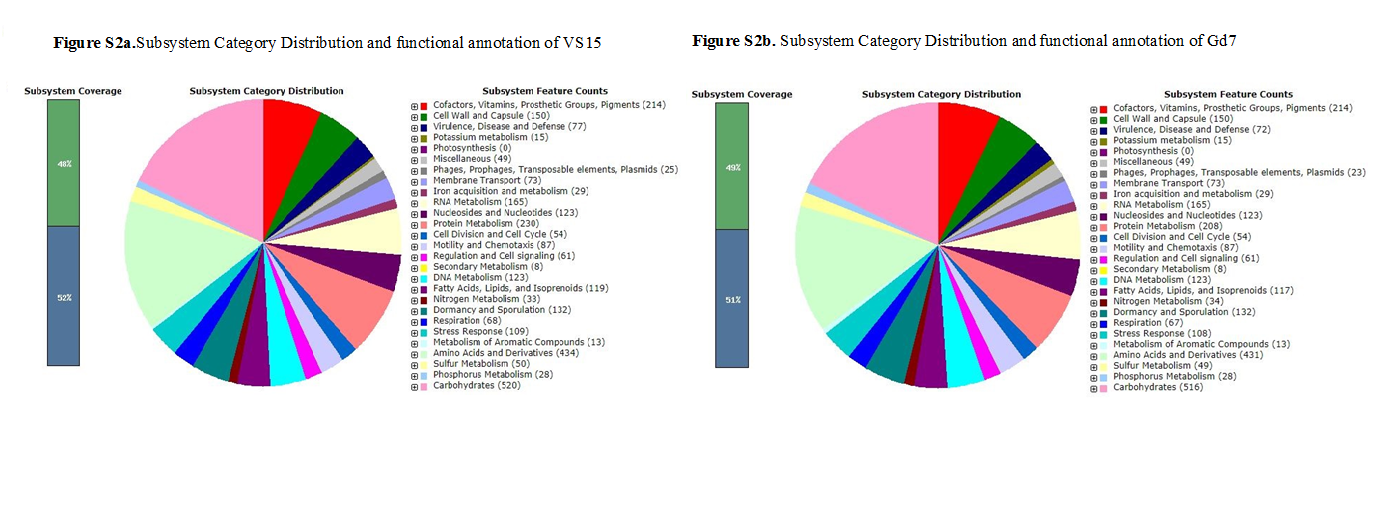

Supplement: Supplementary file 1 [file ijms-21-01299-s001.zip › supplementary/Supplementary_figures_S2A___S2b.tif]

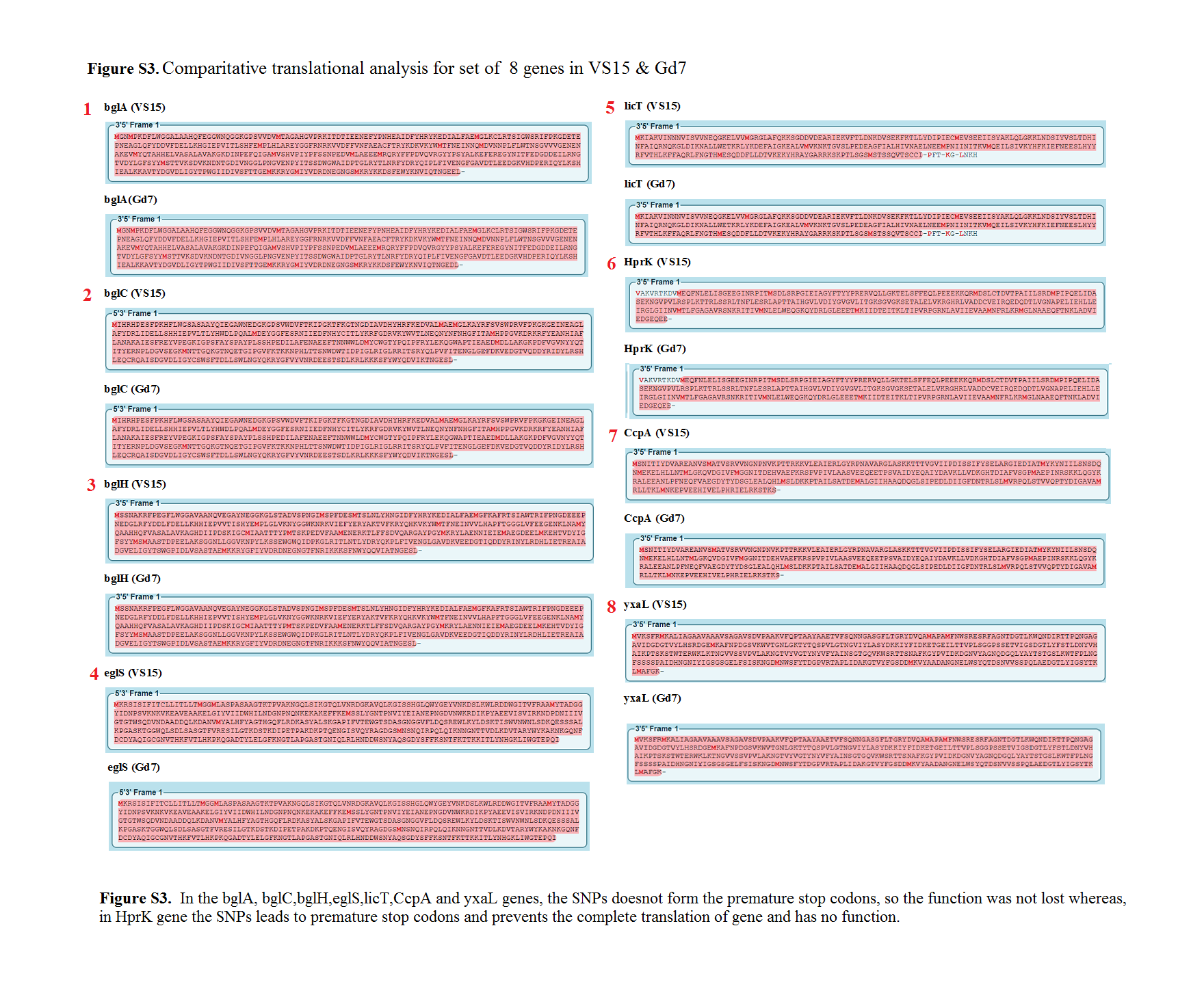

Supplement: Supplementary file 1 [file ijms-21-01299-s001.zip › supplementary/Supplementary_figure_S3.tif]
